# Lessons Learnt From COVID-19 Vaccine Hesitancy: The Role of Culturally Concordant Providers

**DOI:** 10.7759/cureus.102726

**Published:** 2026-01-31

**Authors:** Kalpana P Padala, Ivy McGeeBlanks, Prasad R Padala

**Affiliations:** 1 Geriatric Research Education and Clinical Center, Central Arkansas Veterans Healthcare System, Little Rock, USA; 2 Primary Care Service, Central Arkansas Veterans Healthcare System, Little Rock, USA

**Keywords:** covid-19, minorities, operation warp speed, vaccine hesitancy, world health organization

## Abstract

Vaccine uptake during the pandemic had varied widely within the US with the lowest rates of vaccine uptake in predominantly southern states. While vaccine hesitancy is prevalent in the public, higher rates of vaccine hesitancy are seen among African Americans and Hispanics. The discrepant vaccine hesitancy is salient since minorities have been particularly hit hard by the pandemic with disproportionately higher rates of infection and morbidity. Recent data show disparities in vaccination rates among minorities in some parts of the US showing White residents to be twice as likely to receive the COVID-19 vaccine compared to their Black counterparts. This perspective discusses factors related to higher vaccine hesitancy among minorities and outlines the experience of a culturally concordant physician champion improving vaccine uptake at an academic medical center. Lessons from the last pandemic might help us better prepare for future ones and cope with a changing landscape that fosters political influence on vaccine uptake. Based on the lessons, we propose a new model for vaccine hesitancy, the 6C model.

## Editorial

COVID-19 vaccine development has been a great success story of our times. Operation Warp Speed had invested Billions of dollars towards the efforts on vaccine development in an unprecedented public-private partnership. Pfizer/BioNTech was the first vaccine to obtain emergency use authorization (EUA) in the UK and the US. This is quite a triumph, to develop, test, and get FDA approvals within 10 months of the pandemic, a process that would have taken more than a decade under usual circumstances [1]. Moderna was the second vaccine to receive EUA, followed by the single-dose Janssen vaccine. However, there have been significant challenges in distribution and mass vaccination with widespread variation between different countries and ethnicities. As of May 21, 2021, 127 million Americans have been fully vaccinated, amounting to 39% of the US population. In contrast, only 4.9% of the world population is fully vaccinated [2]. Within the US, there is wide variation as well with states in the North East, such as Maine, have over half the population fully vaccinated, whereas the southern states, such as Mississippi, have just over a quarter of the population fully vaccinated [3]. Some of these variations could be explained by the demographic and ethnic composition of the states.

While the COVID vaccines were being developed, there was mixed support in the public for the same. A survey of over 1000 US adults showed that only 49% were willing to take a vaccine developed for COVID, much less than the 70% needed for achieving herd immunity [4,5]. Interestingly, in the same survey, while 20% said that they would not take the vaccine, the other 30% were unsure [4]. These middle 30% could potentially be persuaded by messaging either from science or anti-vaccine activists. As the COVID vaccines became available for the public, vaccine hesitancy and refusal loomed as major problems as anti-vaccine activists gained traction. Vaccine hesitancy is defined as the delay or refusal to be vaccinated despite its availability. This complex issue is both context and culture-specific. Vaccine use behavior is well described by the 5C model. The 5C model uses the health behavior model and the theory of planned behavior to break down vaccine hesitancy into distinct categories of a) complacency, b) convenience, c) confidence, d) calculation (risk vs. benefit), and e) collective responsibility [6].

Vaccine hesitancy in the war against COVID

If the development of the vaccines was a monumental task, getting them into the arms of a critical mass of people to generate herd immunity was an equally challenging task. The World Health Organization describes vaccine hesitancy as one of the top health threats globally [7]. Vaccine hesitancy preceded the COVID-19 pandemic in the US and is on the rise during the pandemic, fueled by myths and misconceptions, conspiracy theories, and statements from antivax groups. Two recent studies of nationally representative samples found the vaccine hesitancy for COVID vaccines ranging from 22-25% in the US [8,9]. Even more alarmingly, another study (N=1878) found that there was a high prevalence of vaccine hesitancy in African Americans, Hispanics, those with children at home, individuals with lower education and incomes, and those residing in rural areas [8]. This is salient since minorities have been particularly hit hard by the pandemic with disproportionately higher rates of infection and morbidity. Yet, it has been found that minorities are especially concerned about being made “guinea pigs,” and have lower levels of trust in the Government compared to their White counterparts due to prior and ongoing experiences. Disparities in vaccination rates among minorities have also begun to increase, with data in Michigan showing that White residents are twice as likely to receive the COVID-19 vaccine compared to their Black counterparts. Areas such as Detroit and Genesee County, which have some of the highest populations of African Americans in Michigan, have some of the lowest vaccination rates in the state. The concept of “Black Immunity” for COVID has been reported early in the pandemic and may be shifting with the increase in numbers among the minorities [10]. Overcoming vaccine hesitancy, particularly among minorities, is critical, as it was estimated that at least 70% of the US population needs to be vaccinated with the COVID vaccine to achieve herd immunity [11].

Factors related to higher vaccine hesitancy among minorities

Higher rates of vaccine hesitancy among minorities are multifactorial including lower access and interaction with healthcare professionals, historical biomedical and healthcare-related mistrust, lower participation of minorities in clinical trials, and cost-related concerns [12]. Our recent collective effort to reckon the systemic racism in US healthcare has unfortunately picked fresh the wounds dating back to the Tuskegee and the Henrietta Lacks experiences, further alienating minorities. Another important factor that leads to vaccine hesitancy is the low levels of community engagement seen in the COVID-19 vaccine development [12]. Politicization of pandemic response has further diminished trust in the COVID-19 vaccine. Vaccine hesitancy may be reduced by educating the general population and the populations with vaccine hesitancy, investing in building trust, increasing transparency in Government contracts, establishing contracts with minority-based businesses, and engaging a nongovernmental “honest broker” organization to monitor vaccine access [12]. We posit that all factors in the 5C model are either influenced by or need to be informed by cultural sensitivity to bridge the chasm of vaccine hesitancy among minorities. One such important factor in patient acceptance of a vaccine intervention is providing unequivocal recommendation(s) from a culturally concordant healthcare provider. Hence, we discuss the role of a culturally concordant healthcare provider in improving vaccine uptake among minorities.

Lessons learned at an academic center in vaccine study and reducing hesitancy

Our site participated in one of the COVID vaccine clinical trials as part of the Department of Veterans Affairs’ involvement in Operation Warp Speed. Unprecedented administrative, regulatory, and Public Affairs Service support was offered to our team. Our hospital made an entire unit available for the clinical trial, multiple personnel were hired, and others reassigned to the study. Ad-hoc regulatory meetings were conducted to fast-track approvals, incredible support was extended from the Central Office, and significant safety enhancements were quickly accomplished in pharmacy, laboratory, and testing rooms. It truly started at a warp speed. Several tools were made available for recruitment, including traditional tools such as flyers and database queries from a group of participants who had provided consent to be contacted for future studies. Non-traditional recruitment tools, such as the Office of Public Affairs sending out public safety announcements and the Volunteer Service putting together a care package for study participants, were also used. Despite the sponsor cutting back recruitment goals and one of the study team members contracting COVID, recruitment was highly successful with a culturally concordant provider as the driver of recruitment.

Culturally concordant physician champion breaks barriers

A registry of persons interested in participation in a COVID-19 vaccine trial was the first source of recruitment, followed by outreach and education by physician champions. The registry had 11.5% minorities and 30% women. Overall success in recruiting from the registry was 4.5% of the participants contacted. Two physician champions accounted for over half of the recruitment, and their success in recruitment was up to 14.3% of the participants contacted. Overall, 12.5% of minority recruitment was from the registry and self-referral each, while the physician champions accounted for 75% of the minority recruitment. A culturally concordant physician was over 17 times more successful in minority recruitment than another physician champion (14.3% vs. 0.8%), finally accounting for 62.5% of the minorities recruited in the study.

Discussion

While COVID-19 vaccines were developed at a Warp speed, uptake varied widely based on geography and ethnicity. Vaccine hesitancy was disproportionately high among minorities reflecting in lower vaccination rates. One of the factors for higher vaccine hesitancy among minorities is their underrepresentation in clinical trials. The COVID-19 vaccine trials were no exception, with 10% Black and 13% Latinx in the Pfizer/BioNTech study. The database provided by a reputable national recruitment site only had 11.5% of minorities, although oversampling was employed to obtain a better representation of minorities.

**Figure 1:**
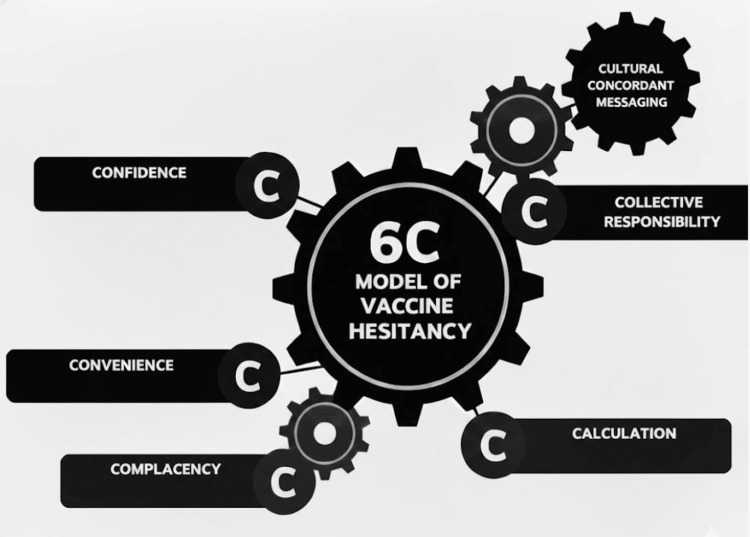
6C model of vaccine hesitancy

Although investigators from minority backgrounds are underrepresented in biomedical research, in our experience, they have a lot of sway in improving minority participation. We posit that all factors in the 5C model of vaccine hesitancy are either influenced by or need to be informed by cultural sensitivity to bridge the chasm of vaccine hesitancy among minorities. We propose a 6C model of vaccine hesitancy with culturally concordant messaging at the top guiding all interventions (Figure 1).

Culturally concordant messaging includes the use of down-to-earth language, strategic self-disclosure, use of open-ended questions, emphasizing safety of the family, and staying away from political discussions and scare tactics. These strategies were used successfully by the physician champions, resulting in higher rates of minority participation at our site. It was evident that the participants banked heavily on the pre-existing trust in the relationship with a culturally concordant provider, as many said, “I’m here because Dr. M told me about this and she would not have told me this, if it were not good for me”.

Albeit from a single site, our experience emphasizes the need for culturally concordant physicians and healthcare providers in improving COVID vaccine uptake among minorities. There have been a lot of creative ways to improve vaccine uptake, including the $1 million lottery offered in the state of Portland, employers providing incentives (both monetary and non-monetary) for vaccination, and celebrities endorsing vaccination. Adding the voice of a culturally concordant physician to these efforts has the potential to further improve vaccine uptake.

Lessons from this report are particularly relevant today as the Centers for Disease Control and Prevention (CDC) recently cut the number of recommended childhood vaccines from 17 to 11 and placed some well-accepted vaccines under “Shared Decision Making” rubric [13]. Having trusting relationships with providers is critical in arriving at medically appropriate decisions. It’s never been more urgent to have culturally concordant physicians at the discussion table.
